# The first data on the innervation of the lophophore in the rhynchonelliform brachiopod *Hemithiris psittacea*: what is the ground pattern of the lophophore in lophophorates?

**DOI:** 10.1186/s12862-017-1029-5

**Published:** 2017-07-31

**Authors:** Elena N. Temereva, Tatyana V. Kuzmina

**Affiliations:** Department of Invertebrate Zoology, Biological Faculty, Moscow State University, Leninskie Gory, 1-12, 119991 Moscow, Russia

**Keywords:** Brachiopoda, Nervous system, Lophophorates, Lophophore, Evolution, Phylogeny

## Abstract

**Background:**

The nervous system in brachiopods has seldom been studied with modern methods. An understanding of lophophore innervation in adult brachiopods is useful for comparing the innervation of the same lophophore type among different brachiopods and can also help answer questions about the monophyly of the lophophorates. Although some brachiopods are studied with modern methods, rhynchonelliform brachiopods still require investigation. The current study used transmission electron microscopy, immunocytochemistry, and confocal laser scanning microscopy to investigate the nerve system of the lophophore and tentacles in the rhynchonelliform *Hemithiris psittacea*.

**Results:**

Four longitudinal nerves pass along each brachium of the lophophore: the main, accessory, second accessory, and lower. The main brachial nerve extends at the base of the dorsal side of the brachial fold and gives rise to the cross nerves, passing through the extracellular matrix to the tentacles. Cross nerves skirt the accessory brachial nerve, branch, and penetrate into adjacent outer and inner tentacles, where they are referred to as the frontal tentacular nerves. The second accessory nerve passes along the base of the inner tentacles. This nerve consists of Ʊ-like parts, which repetitively skirt the frontal and lateral sides of the inner tentacle and the frontal sides of the outer tentacles. The second accessory nerve gives rise to the latero-frontal nerves of the inner and outer tentacles. The abfrontal nerves of the inner tentacles also originate from the second accessory nerve, whereas the abfrontal nerves of the outer tentacles originate from the lower brachial nerve. The lower brachial nerve extends along the outer side of the lophophore brachia and gives rise to the intertentacular nerves, which form a T-like branch and penetrate the adjacent outer tentacles where they are referred to as abfrontal nerves. The paired outer radial nerves start from the lower brachial nerve, extend into the second accessory nerve, and give rise to the lateroabfrontal tentacular nerves of the outer tentacles.

**Conclusions:**

The innervation of the lophophore in the rhynchonelliform *Hemithiris psittacea* differs from that in the inarticulate *Lingula anatina* in several ways. The accessory brachial nerve does not participate in the innervation of the tentacles in *H. psittacea* as it does in *L. anatina*. The second accessory nerve is present in *H. psittacea* but not in *L. anatina*. There are six tentacular nerves in the outer tentacles of *H. psittacea* but only four in all other brachiopods studied to date. The reduced contribution of the accessory brachial nerve to tentacle innervation may reflect the general pattern of reduction of the inner lophophoral nerve in both phoronids and brachiopods. Bryozoan lophophores, in contrast, have a weakened outer nerve and a strengthened inner nerve. Our results suggest that the ancestral lophophore of all lophophorates had a simple shape but many nerve elements.

## Background

The Lophophorata has traditionally been considered a monophyletic superphylum that includes three phyla: the Brachiopoda, Phoronida, and Bryozoa (=Ectoprocta) [1–3]. Although the monophyly of the lophophorates has been challenged by recent molecular data [4–7], it has also been supported by other molecular data [8, 9] and by recent morphological studies [10, 11]. It follows that determining whether the lophophorates are monophyletic will require additional molecular and morphological data including new morphological data on the lophophore, which is a tentacular organ possessed by all lophophorates. Previous research has indicated that analysis of lophophore organization can provide insights into the phylogeny of the lophophorates [10, 11].

Adult brachiopods are benthic, marine animals. There are three main groups of brachiopods: Linguliformea, Craniiformea, and Rhynchonelliformea [12, 13]. These groups differ in various characteristics including the organization of the shell. According to the chemical structure of the shell, brachiopods can be divided into a group with organophosphatic shells (Linguliformea) and groups with calcareous shells (Craniiformea and Rhynchonelliformea). On the other hand, the morphology of the shell with an emphasis on the presence or absence of articulatory structures along the hinge leads to division of brachiopods into two groups: Articulate (= Rhynchonelliformea) and Inarticulate (= Linguliformea + Craniiformea). The relationship between the three main groups of brachiopods is still discussed [14].

Among brachiopods, there are six types of the lophophore organization [15]. The lophophore organization can differ between species within the same group and can be similar between species of different groups. The widest diversity of types of the lophophore organization occurs within Rhynchonelliformea [15].

The organization of the nervous system in brachiopods has usually been investigated via light microscopy [16–19]. There are, however, some transmission electron microscopy (TEM) data on the innervation of brachiopod tentacles [20–22]. The most recent results, which were obtained by confocal laser scanning microscopy (CLSM) and immunocytochemistry, concerned the nervous system of the lophophore and tentacles in the inarticulate brachiopod *Lingula anatina* (Lamark, 1801), which has spirolophe type of lophophore [10]. It will now be useful to compare the innervation of the same type of the lophophore in different species from different taxonomic groups: i.e., the spirolophe lophophore, which is present in inarticulate *Lingula anatina* with organophosphatic shell and in articulate rhynchonelliform brachiopods. The information obtained should increase our understanding of how the lophophore has evolved in brachiopods in particular and in the lophophorates in general.

The recent study provides new data on the innervation of the lophophore in the rhynchonelliform brachiopod *Hemithiris psittacea* (Gmelin, 1791). The new data are then used to make inferences on the relationships among the Brachiopoda, Phoronida, and Bryozoa.

## Results

### Morphology of the lophophore and tentacles in *H. psittacea*

**Fig. 1:**
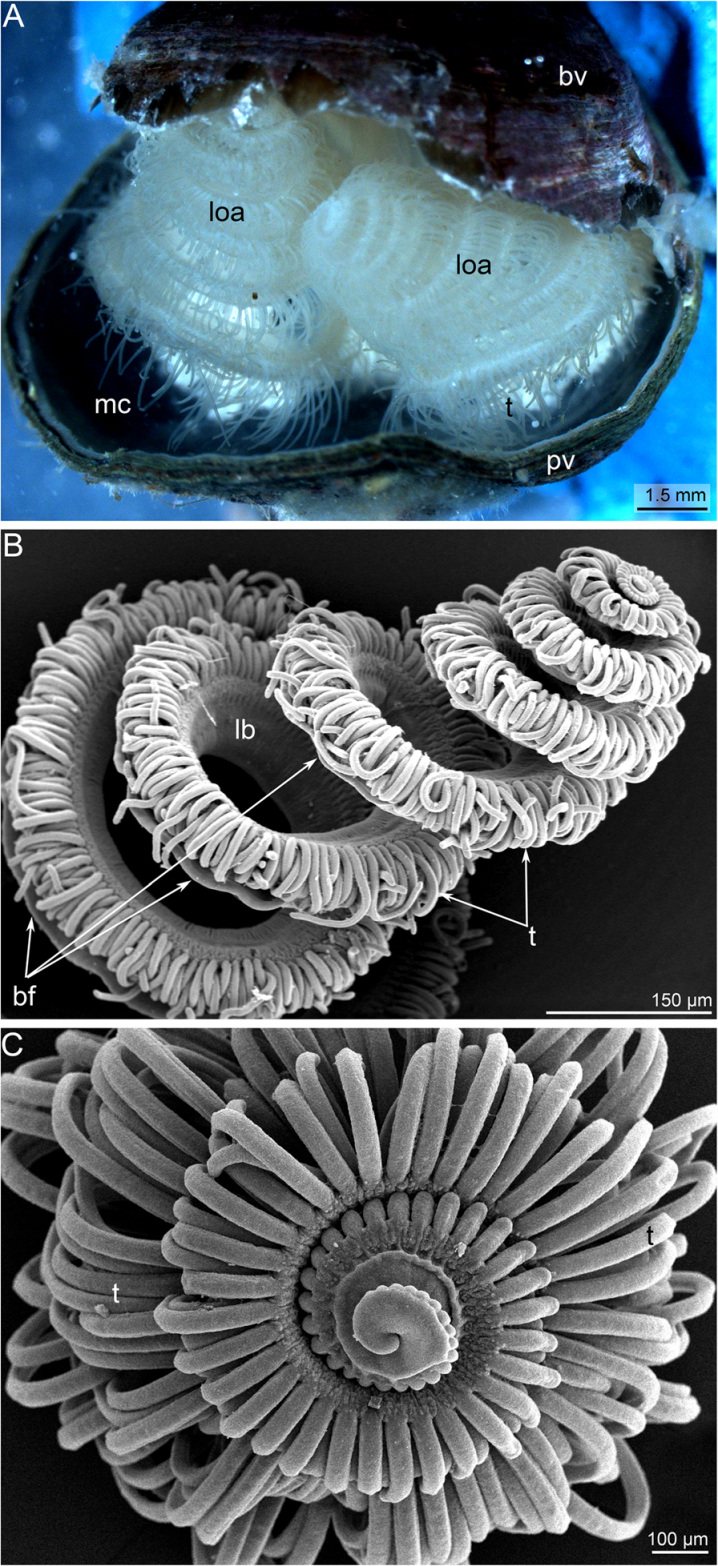
Organization of the lophophore in *Hemithiris psittacea*. **a** Photograph of a live animal: the brachial valve (bv) is broken. Two arms of the spirolophe lophophore are visible. **b** A brachium; SEM; lateral view. **c** The distal end of the brachium with the youngest tentacles; SEM; top view. Abbreviations: bf – brachial fold; bv – brachial valve of shell; lb – lophophore base; loa – lophophore arm (brachium); mc – mantle cavity; pv – pedal valve of shell; t – tentacle

**Fig. 2:**
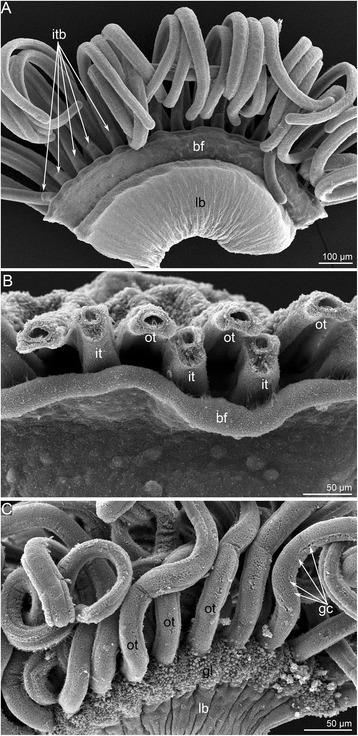
Details of lophophore organization in *Hemithiris psittacea*; SEM. **a** A portion of the lophophore brachium, viewed from the brachial fold. **b** Two rows of tentacles (inner and outer) and the brachial fold, viewed from the top. **c** The base of the lophophore arm, viewed from the outer side. The layer of glandular cells is visible (gl). Abbreviations: bf – brachial fold; gc – openings of glandular cells along the lateroabfrontal side of tentacle; gl – layer of glandular cells at the base of the outer side of the lophophore; it – inner tentacle; itb – swollen base of inner tentacles; lb. – lophophore base; ot – outer tentacle

**Fig. 3:**
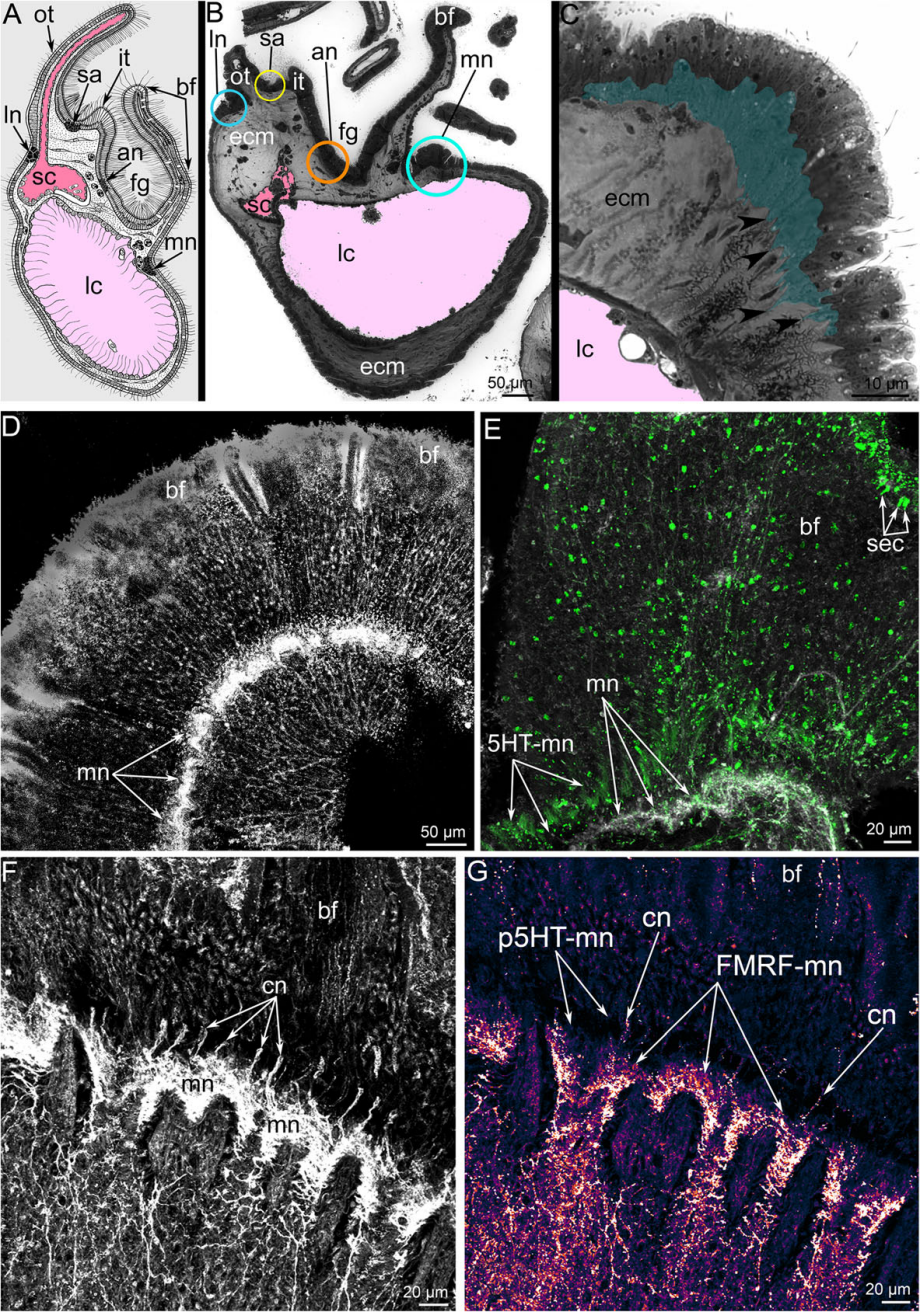
Location of brachial nerves in the brachium and structure of the main brachial nerve in *Hemithiris psittacea*. **a** Schematic cross section of the lophophore brachium. **b** Semi-thin cross section of the brachium. The location of different nerves is marked by circles of different colors, which correspond to the colors of nerves in Fig. 5. **c** Semi-thin section of the epithelium, which contains the main brachial nerve (marked by color). The cross nerves, which originate from the main nerve, are indicated by arrowheads. **d** Z-projections of the brachial fold with the main brachial nerve at the base. Immunostaining against alpha acetylated-tubulin. **e** Z-projections of the brachial fold: immunostaining against alpha acetylated-tubulin (grey) and serotonin (green). The sensory cells are visible along the edge of the brachial fold. **f** Z-projections of the base of brachial fold: immunostaining against alpha acetylated-tubulin. The main nerve and a portion of the cross nerves (cn) are visible. **g** Z-projections of the base of brachial fold: immunostaining against FMRFamide. The empty space, which corresponds to the location of serotonin-like immunoreactive elements of the main nerve, is visible (p5HT-mn). Abbreviations: 5HT-mn – serotonin-like immunoreactive elements of the main brachial nerve; an – accessory brachial nerve; bf – brachial fold; cn – cross nerves; ecm – extracellular matrix; fg – food groove; FMRFamide-mn – FMRFamide-like immunoreactive elements of the main brachial nerve; it – inner tentacle; lc – large canal of the lophophoral coelom; ln – lower brachial nerve; mn – main brachial nerve; ot – outer tentacle; sa – second accessory nerve; sc – small canal of the lophophoral coelom; sec – sensory cells

**Fig. 4:**
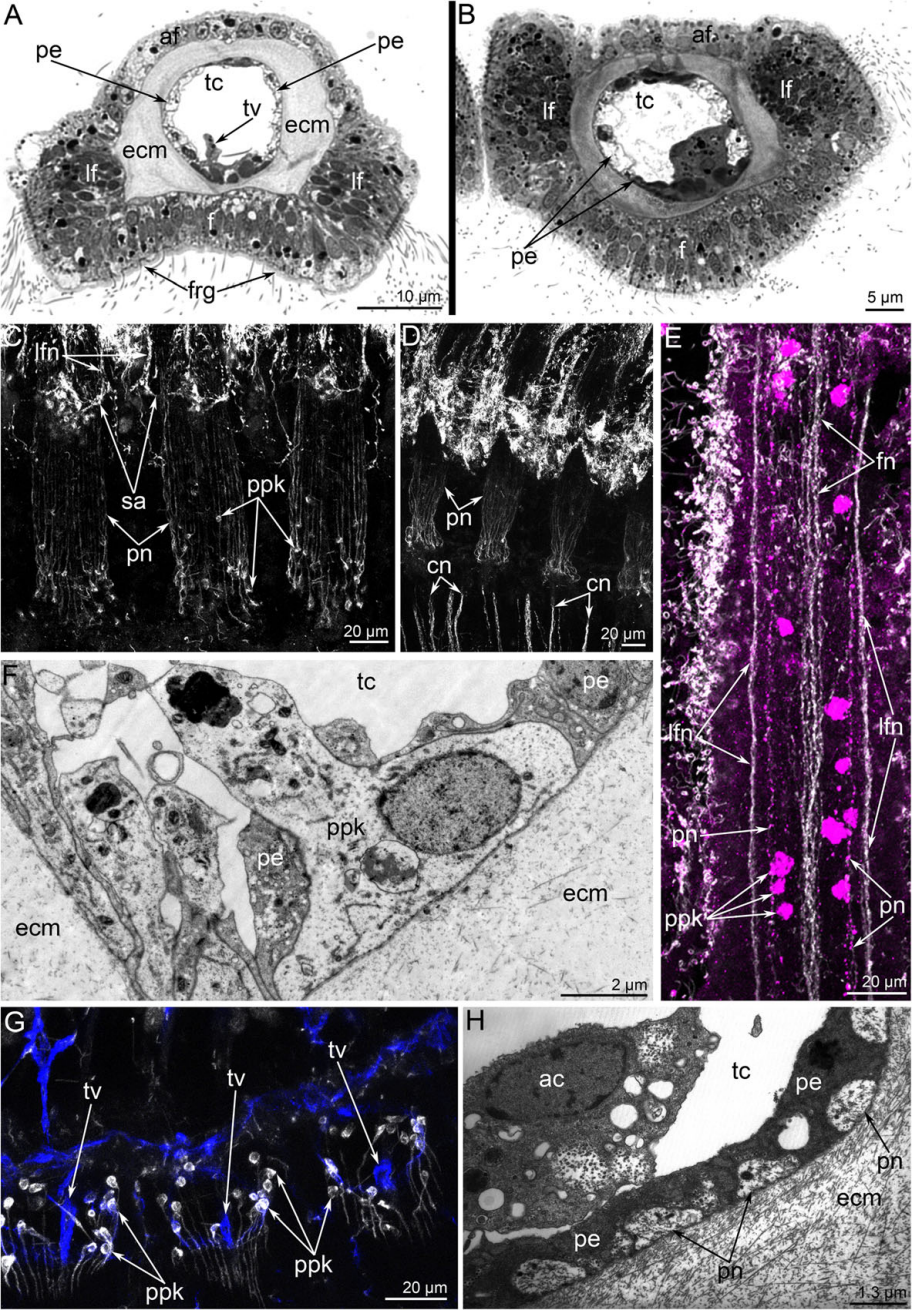
Organization of tentacles in *Hemithiris psittacea*. Semi-thin cross sections (**a**, **b**); Z-projections after mono- (**c**, **d**) and double- (**e**) immunostaining against alpha acetylated-tubulin (grey) and serotonin (magenta); and staining with phalloidin (blue) (**g**); thin sections (**f**, **h**). **a** Outer tentacle. **b** Inner tentacle. **c** Z-projection of peritoneal nerves in outer tentacles. **d** Z-projection of peritoneal nerves in inner tentacles. **e** Z-projection of inner tentacle. **f** Cross section of the base of an outer tentacle: the large cell (ppk) with electron-lucent cytoplasm is visible. **g** Z-projection of the base of outer tentacles: perikarya of peritoneal cells (ppk) are visible. **h** Cross section of the lateral side of an inner tentacle: large peritoneal neurites (pn) contain electron-lucent cytoplasm. Abbreviations: ac – amoebocyte; af – abfrontal zone; cn – cross nerve; ecm – extracellular matrix; f – frontal zone; fn – frontal tentacular nerve; frg – frontal groove; lf – laterofrontal zone; lfn – laterofrontal tentacular nerve; pe – peritoneal cell of coelomic lining; pn – peritoneal neurite; ppk – peritoneal perikarya; sa – second accessory nerve; tc – tentacular coelom; tv – tentacular blood vessel

**Fig. 5:**
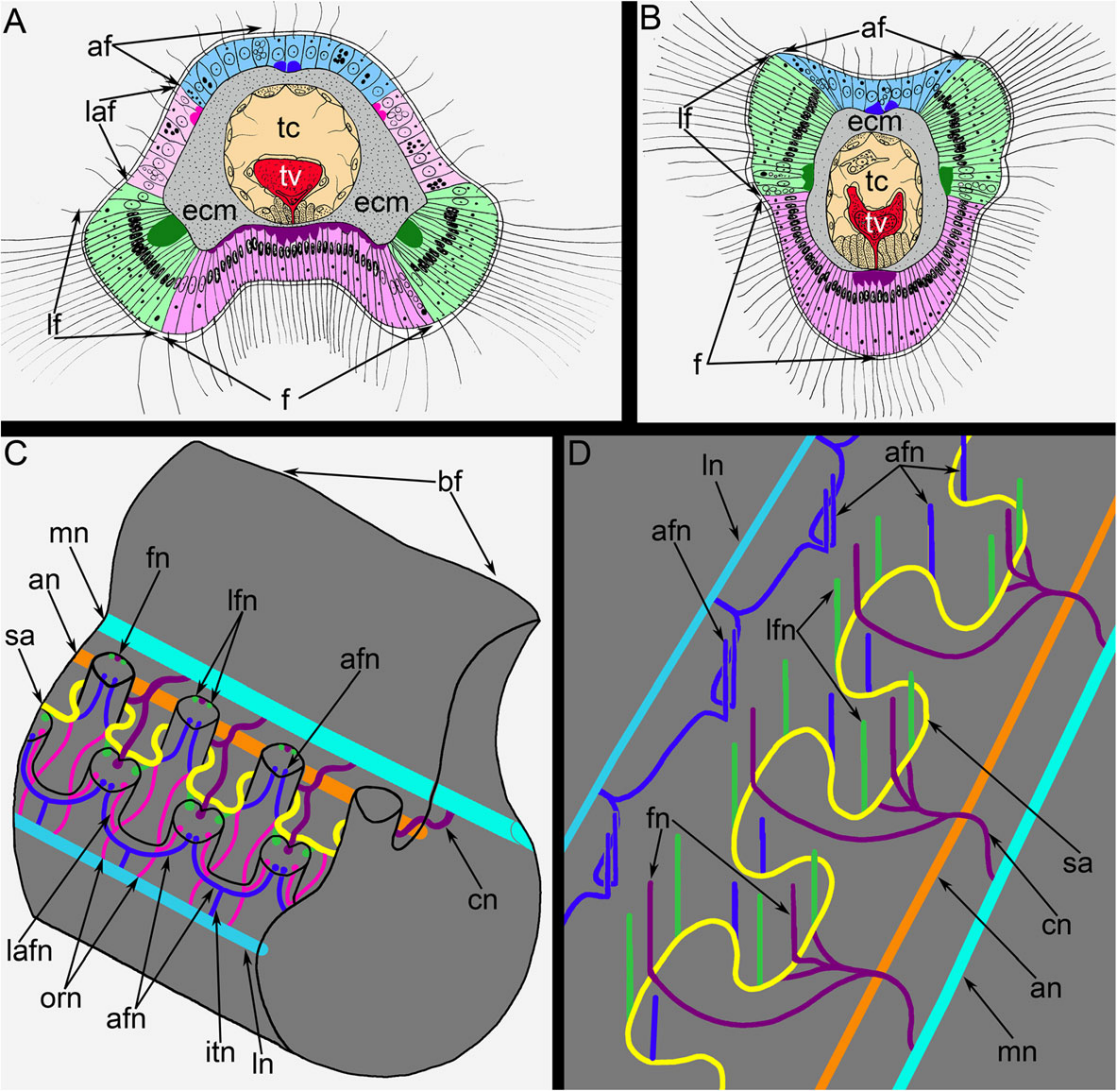
Schemes of innervation of tentacles and the lophophore in *Hemithiris psittacea*. **a** Organization of the outer tentacles. **b** Organization of the inner tentacles. **c** A portion of the lophophore brachium with different nerve tracts. **d** The scheme of the lophophore nervous system: tentacles, brachial fold, and paired outer radial nerves are not shown. Abbreviations: af – abfrontal zone; afn – abfrontal tentacular nerve; an – accessory brachial nerve; cn – cross nerve; ecm – extracellular matrix; f – frontal zone; fn – frontal tentacular nerve; itn – intertentacular nerve; laf – laterofrontal zone; lafn – lateroabfrontal tentacular nerve; lf – laterofrontal zone; lfn – laterofrontal tentacular nerve; ln – lower brachial nerve; mn – main brachial nerve; orn – paired outer radial nerves; sa – second accessory nerve; tc – tentacular coelom; tv – tentacular blood vessel

*H. psittacea* has a shell that consists of a dorsal (=brachial) and ventral (= pedicle) valve (Fig. 1a). *H. psittacea* possesses a spirolophe lophophore, which has two arms with a mouth in between (Figure 1a). The distal ends of the brachial axes are twisted into spirals (Figure 1a, b). The youngest tentacles are located at the distal end of each brachium (Figure 1c). The lophophore nears a brachial fold (Figure 2a). The brachial fold repeats the shape of the lophophore and covers the tentacle bases (Figs. 1b, 2a). The food groove extends between the brachial fold and tentacle bases and then passes into the mouth (Figs. 3a, b). There are two rows of tentacles: inner, which is located near the brachial fold, and an outer, external row (Figure 2b). Tentacles in both rows are located in antiphase (Figure 2b). Several zones along both rows of tentacles differ from each other in fine structure of the integument (Fig. 4a, b). There are four zones around each tentacle: one frontal, two latero-frontal, and one abfrontal (Fig. 5a, b). The frontal zone is the closest zone to the brachial fold; the abfrontal zone is opposite the frontal zone. Tentacles of the inner and outer rows differ from each other in the shape of the cross section (Fig. 4a, b). Tentacles of the outer row have a ciliated groove, which extends along the frontal side (Figs. 4a, 5a). Inner tentacles have glandular swellings at the base of the frontal side (Figure 2a). The bases of the outer tentacles are also covered by numerous glandular cells (Figure 2c).

### Morphology of the lophophore nervous system

Four longitudinal nerves extend along each brachium of the lophophore: the main brachial nerve, the accessory brachial nerve, the second accessory nerve, and the lower brachial nerve.

The main brachial nerve extends along the external side of the base of the brachial fold (Fig. 3a-c). The cross nerves originate every 10 μm from the main brachial nerve and extend into the extracellular matrix (ECM) of the brachium (Figure 3f). The accessory brachial nerve passes along the inner side of the food groove (Figure 3b). The cross nerves skirt the accessory brachial nerve and are not connected with it (Figure 5c). Each cross nerve branches into two nerves, which pass to the second accessory nerve. Some neurites of cross nerves extend into and become part of the second accessory nerve, whereas other neurites transform into the frontal tentacular nerves of the inner and outer tentacles (Figure 5d). The second accessory nerve extends into the epithelium at the base of tentacles between them and consists of Ʊ-shaped parts, which repetitively skirt the frontal and lateral sides of the inner tentacles and pass along the frontal sides of the outer tentacles (Figs. 3a, b; 5c, d). The latero-frontal and abfrontal nerves of the inner tentacles and the latero-frontal nerves of the outer tentacles originate from the second accessory nerve. The lower brachial nerve extends along the outer side of each brachium and gives rise to the abfrontal nerves of the outer tentacles (Fig. 3a, b). The lower brachial nerve gives rise to the intertentacular nerves, which branch and penetrate adjacent outer tentacles, where they are referred to as abfrontal tentacular nerves (Fig. 5c, d). The lower brachial nerve connects to the second accessory nerve via the paired outer radial nerves, which extend into the epithelium of the outer side of the brachium and pass between the outer tentacles. These nerves give rise to the latero-abfrontal nerves of the outer tentacles (Figure 5c).

### Immunoreactivity and ultrastructure of the lophophore nerve elements in *H. psittacea*

**Fig. 6:**
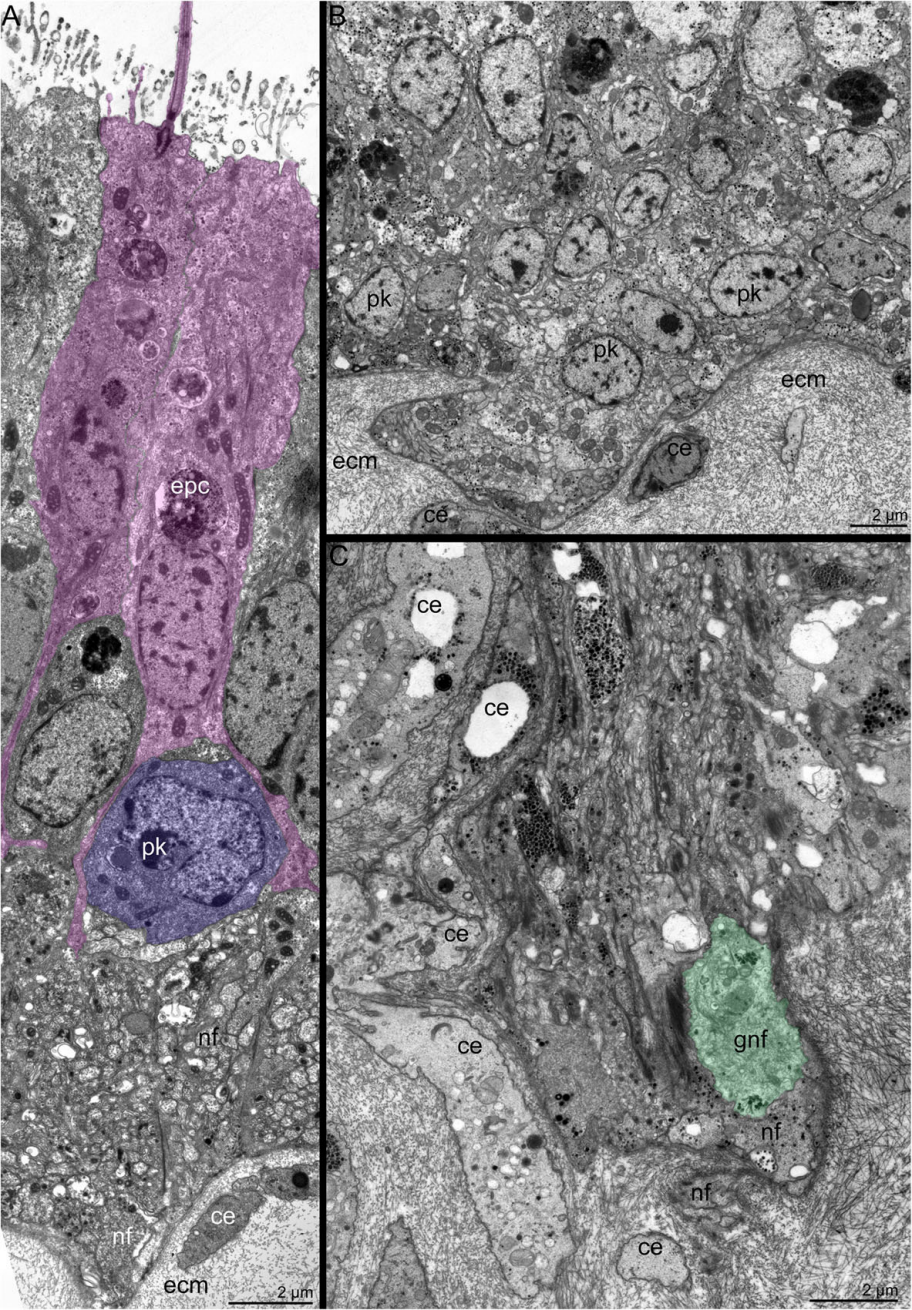
Ultrastructure of the main brachial nerve in *Hemithiris psittacea*. **a** General view of the epithelium, which contains perikarya and neurites of the main brachial nerve. Epithelial cells (pink) form projections, which surround perikarya (violet). **b** Aggregation of perikarya, which are located at the base of the main brachial nerve. **c** Basal portion of the neuropil: aggregation of neurites, including giant nerve fiber (gnf, marked by green). Abbreviations: ce – cell in extracellular matrix; ecm – extracellular matrix; epc – epidermal cell; gnf – giant nerve fiber; pk – perikaryon; nf – nerve fibers

The **main brachial nerve** is the thick nerve that extends at the base of the brachial fold and consists of many neurites, which extend between epithelial cells (Fig. 3c–f). Most neurites of the main brachial nerve exhibit FMRFamide-like immunoreactivity (Figure 3g), whereas the neurites and perikarya located above the main aggregation of neurites exhibit serotonin-like immunoreactivity (Figure 3e). A comparison of the serotonin-like (Figure 3e) and the FMRFamide-like (Figure 3g) immunoreactive stained elements reveals the complementarity of the staining and distinct regionalization of these neurites. The main brachial nerve gives rise to many serotonin-like immunoreactive neurites, which are associated with serotonin-like immunoreactive perikarya and innervate the brachial fold (Figure 3e). Serotonin-like immunoreactive perikarya are scattered in the epithelium of the brachial fold and form a sensory line at the edge of the brachial fold (Figure 3e). According to TEM, the epithelium that contains the main nerve also contains monociliar cells, which form thin basal projections that envelope the basal perikarya (Figure 6a). Numerous perikarya form a large basal layer above the aggregation of nerve fibers (Figure 6b). Within the aggregation of nerve fibers, there are giant neurites whose diameters exceed 5 μm (Figure 6c).

**Fig. 7:**
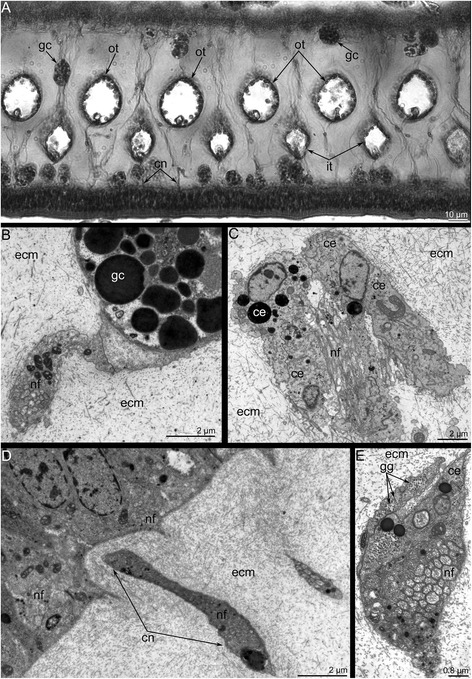
Organization of the cross nerves in *Hemithiris psittacea*. **a** Semi-thin cross section of the base of tentacles. **b** Cross nerve is associated with large globular cell (gc). **c** Cross nerve is surrounded by several cells of extracellular matrix. **d** The cross nerve near the epithelium of the brachial fold. **e** The cross nerve consists of neurites of different diameter and is associated with cell that are filled with glycogenic granules (gg). Abbreviations: ce – cell in extracellular matrix; cn – cross nerve; ecm – extracellular matrix; gc – globular cell; gg – glycogenic granules; it – inner tentacle; nf – nerve fibers; ot – outer tentacle

**Fig. 8:**
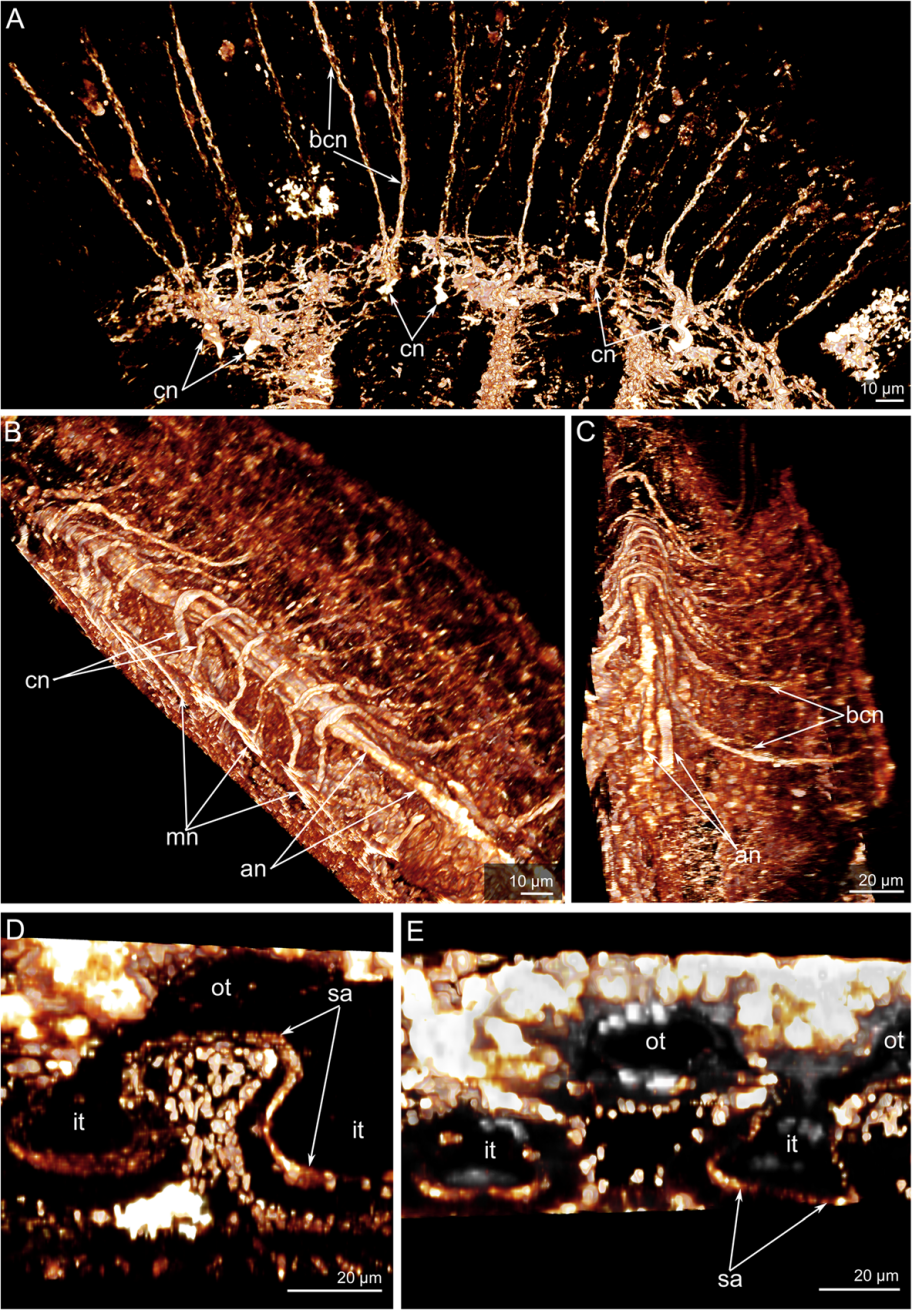
Three-dimensional reconstructions of the cross nerves and the second accessory nerve in *Hemithiris psittacea*. Volume rendering of some nerve elements stained against alpha acetylated-tubulin (glow). **a** The cross nerves (cn) and their branches (bcn) viewed from the brachial fold. **b** The cross nerves viewed from the top. Cross nerves skirt the accessory brachial nerve. **c** The cross nerves and the accessory brachial nerve viewed from the longitudinal axis of the brachium. **d** A portion of the second accessory nerve. **e** A portion of the second accessory nerve: volume rendering of nerve elements (glow), combined with volume rendering of elements stained with phalloidin (grey). Abbreviations: bcn – branches of cross nerves; cn – cross nerve; it – inner tentacle; mn – main brachial nerve; ot – outer tentacle; sa – second accessory nerve

The main brachial nerve gives rise to the **cross nerves**, which extend into the ECM. These nerves are evident in semi-thin sections (Figs. 3c, 7a) and become evident with staining against acetylated alpha tubulin (Figs. 3f, 8a). Some neurites of cross nerves exhibit FMRFamide-like immunoreactivity (Figure 3g). The extensive ECM at the base of each tentacle is penetrated by many tracts, which are formed by cross nerves associated with globular cells (Figure 7a). Each cross nerve skirts the accessory brachial nerve (Fig. 8b, c) and extends to the base of the tentacles, where it bifurcates (Figure 8a) and passes to the adjacent outer and inner tentacles (Figure 5d). According to TEM, the cross nerves are usually associated with large globular cells scattered in the ECM. These cells are characterized by their large size and their numerous, large, electron-dense granules (Figure 7b). In some cases, the cross nerves are associated with cells that are abundant in the ECM and that form an envelope around the neurite bundles (Fig. 7c, e). These envelope cells are rich in glycogenous granules and probably provide nutrition for the nerve fibers (Figure 7e). The cross nerves vary in diameter from 1.5 to 4.5 μm (Fig. 7d, e).

**Fig. 9:**
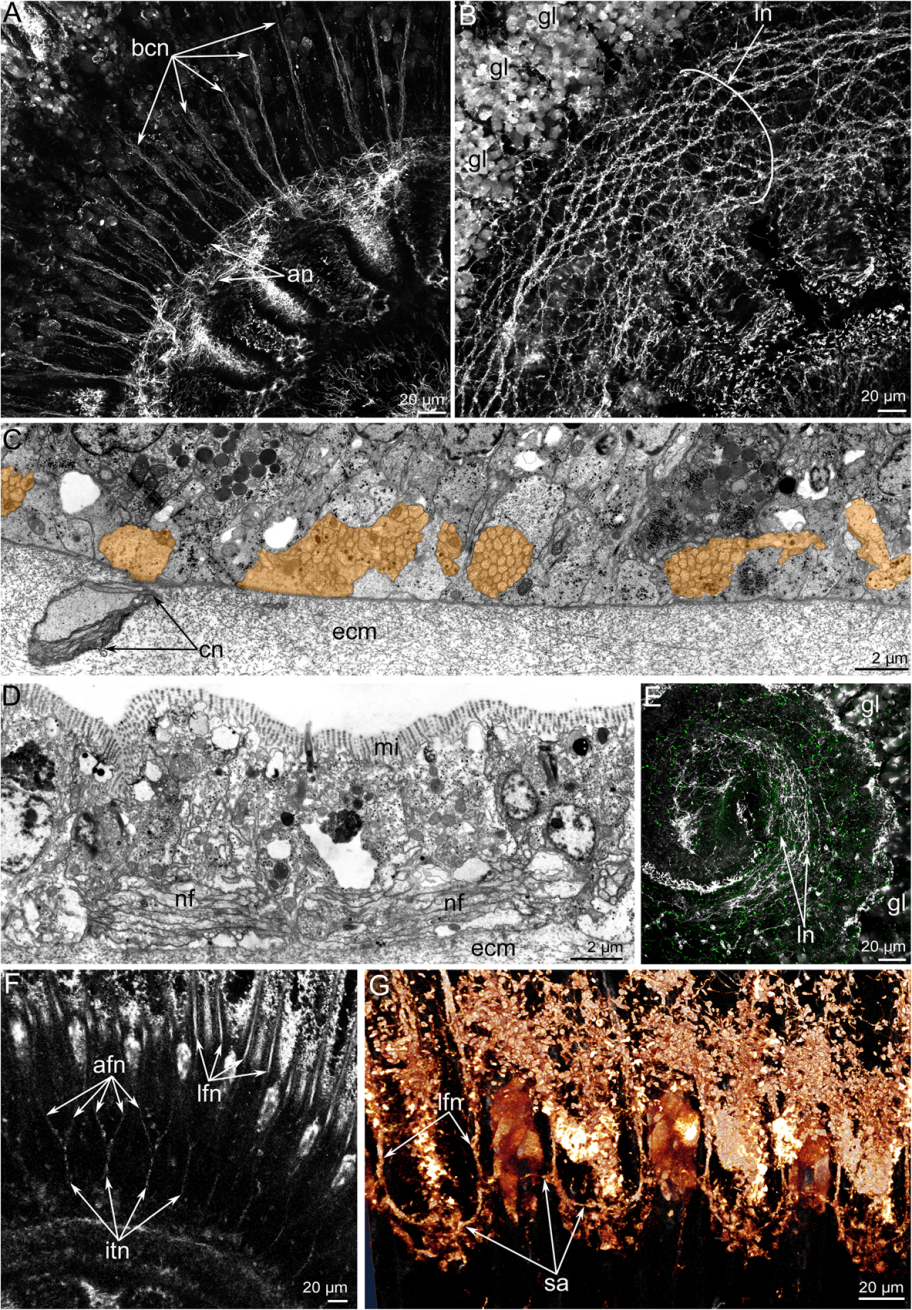
Details of the organization of the accessory brachial nerve, the lower brachial nerve, and the second accessory nerve in *Hemithiris psittacea*. Z-projections after mono- (**a**, **b**) and double- (**e**) immunostaining against alpha acetylated-tubulin (grey) and serotonin (green); volume rendering of some nerve elements stained against alpha acetylated-tubulin (grey – **f**; glow – **g**): thin cross sections of the brachium (**c-d**). **a** The structure of the accessory brachial nerve appears disorganized. **(B)** The lower brachial nerve. **c** Ultrastructure of the accessory brachial nerve, which consists of several neurite bundles (marked by orange) scattered in the epithelium of the food groove. **d** Ultrastructure of the lower brachial nerve consisting of mostly longitudinal neurites. **e** The distal end of the brachium: a mesh of neurites of the lower brachial nerve (ln), which lacks a prominent serotonin-like immunoreactive portion. **f** Intertentacular nerves (itn) originate from the lower brachial nerve and give rise to the abfrontal tentacular nerves (afn) of outer tentacles. **g** A portion of the second accessory nerve (sa). Abbreviations: afn – abfrontal tentacular nerve; an – accessory brachial nerve; bcn – branches of cross nerves; ecm – extracellular matrix; gl – layer of glandular cells; itn – intertentacular nerve; lfn – laterofrontal tentacular nerve; ln – lower brachial nerve; mf – nerve fibers; mi – microvilli; sa – second accessory nerve

The **accessory brachial nerve** is represented by the net of numerous thin neurites that do not connect with the cross nerves (Figure 9a). According to volume rendering of image stacks, the accessory brachial nerve does not contribute to the innervation of tentacles (Fig. 8b, c). The accessory nerve consists of several neurites bundles, which are scattered in the epithelium of the food groove (Figure 9c).

The **second accessory nerve** extends at the base of the tentacles and consists of repetitive Ʊ-like parts (Figs. 5d, 8d, e). This nerve can be visualized by staining against acetylated alpha tubulin. The second accessory nerve connects to the cross nerves and includes their neurites. The second accessory nerve almost completely surrounds the base of each inner tentacle, forming the horseshoe-shaped nerve tract, and passes along the frontal surface of each outer tentacle (Figs. 5d, 8d, e). In volume rendering, the second accessory nerve usually looks like a bridge between the tentacular nerves at the base of each tentacle (Figure 9g). Because of this shape, the second accessory nerve contributes much more to the innervation of the inner tentacles than to the innervation of the outer tentacles.

The **lower brachial nerve** extends along the outer side at the base of each lophophore brachium. According to immunocytochemistry, the lower nerve is represented by a complex net of neurites and thick neurite bundles, which anastomose with each other (Figure 9b). The lower nerve does not exhibit prominent serotonin-like immunoreactivity. Serotonin-like immunoreactive neurites and perikarya are located in the epithelium of the outer side of the brachium (Figure 9e). At the end of each brachium, the lower nerve becomes thinner and looks like an amorphous net of neurites (Figure 9e). In cross sections through the lophophore brachium, most neurites of the lower nerve are cut longitudinally (Figure 9d). Neurites are grouped into bundles with 5–8 neurites per bundle.

The **intertentacular nerves** emanates from the lower brachial nerve (Figure 9f). At the base of the tentacles, each intertentacular nerve branches and gives rise to neurite bundles that penetrate into adjacent outer tentacles as one of two abfrontal neurite bundles.

**Fig. 10:**
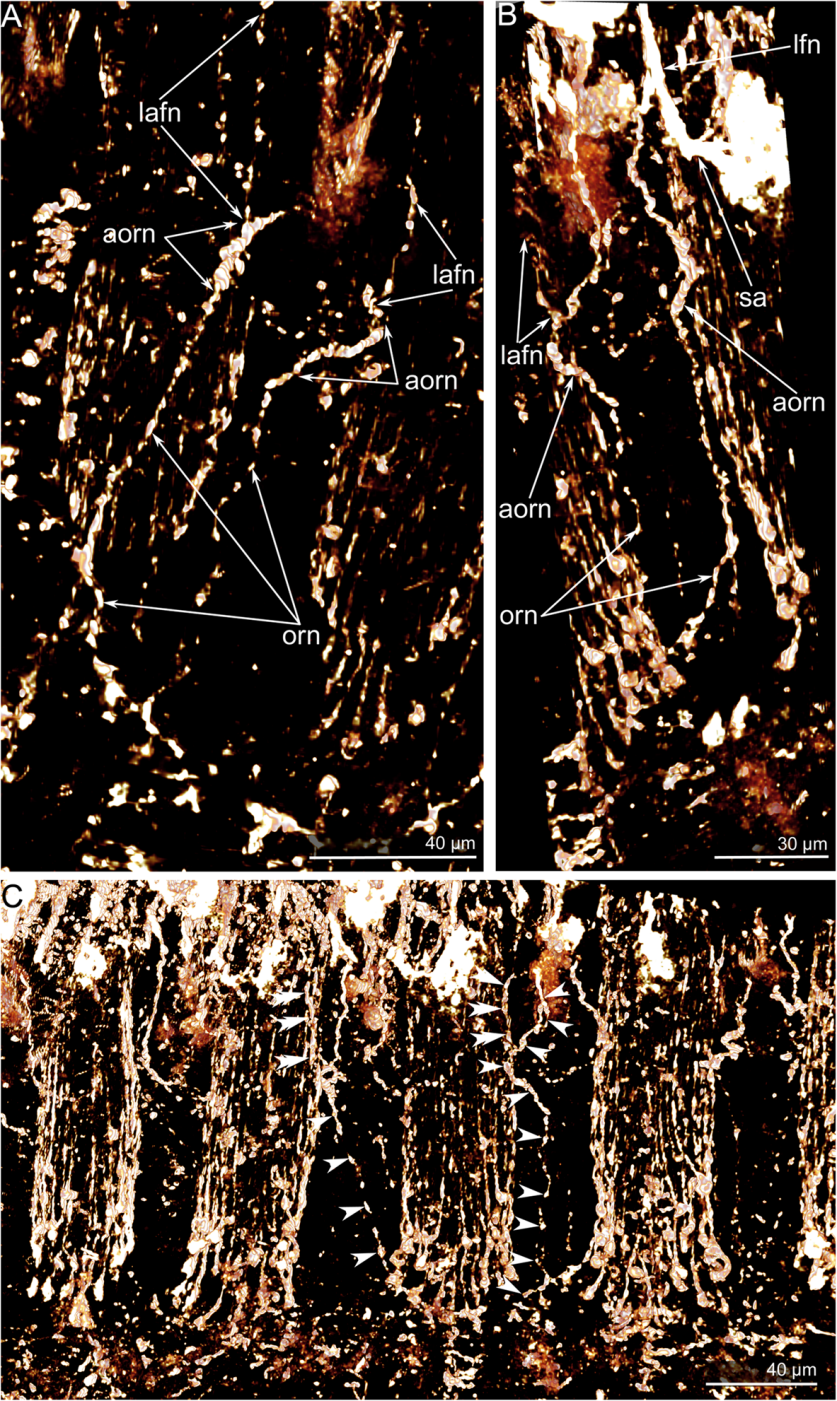
Organization of paired outer radial nerves in *Hemithiris psittacea*. Volume rendering of nerve elements after staining against alpha acetylated-tubulin. **a** Nerves viewed from the outer side of the brachium. **b** Nerves viewed from the inner side of the brachium. **c** A portion of brachium with cylinders of peritoneal neurites of outer tentacles and paired outer radial nerves (marked by arrowheads), which pass between the outer tentacles and skirt them, forming arches (double arrowheads). Abbreviations: afn – abfrontal tentacular nerve; aorn – arch of paired outer radial nerves; lafn – lateroabfrontal tentacular nerve; lfn – laterofrontal tentacular nerve; orn – paired outer radial nerve; sa – second accessory nerve

The lower brachial nerve connects to the second accessory nerve via the **paired outer radial nerves** (Figure 5c). These nerves extend in the epithelium of the brachium between the outer tentacles; each nerve forms an arch (Figure 10a), which closely passes the base of the outer tentacle. Here, each outer radial nerve gives rise to the lateroabfrontal tentacular nerve (Figure 10b) and passes to the second accessory nerve (Figure 10c).

### Innervation of tentacles in *H. psittacea*

Tentacles of both the inner and the outer row are mostly innervated by the second accessory nerve.

**Fig. 11:**
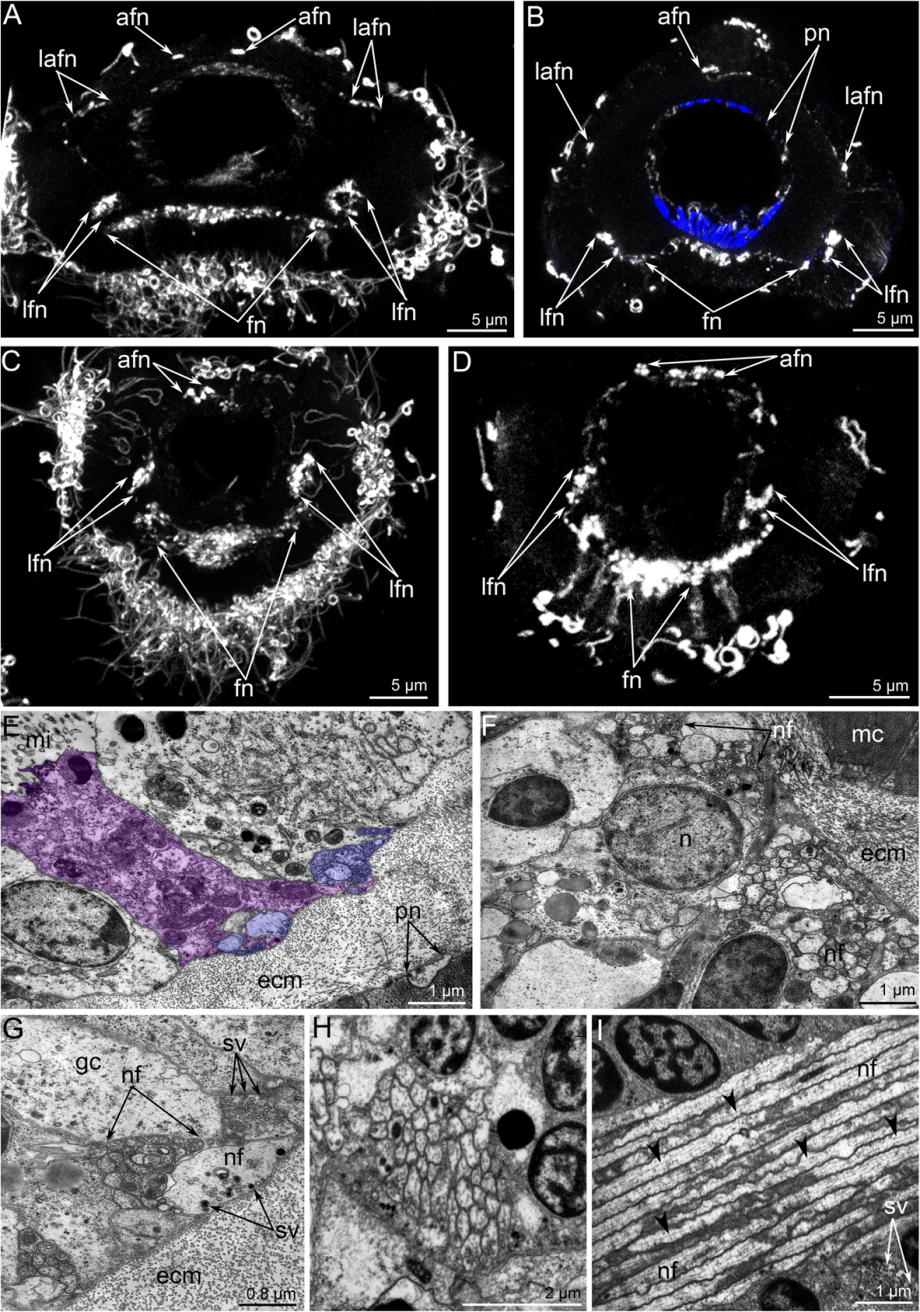
Innervation of tentacles of *Hemithiris psittacea*. Z-projections after immunostaining against alpha acetylated-tubulin – grey (**a**, **c-d**), against alpha acetylated-tubulin and with phalloidin – blue (**b**); thin cross section (**e-h**) and longitudinal section (**i**) of tentacles. **a** Optical cross section of outer tentacle at its base. **b** Optical cross section of outer tentacle near its tip. **c** Optical cross section of inner tentacle at its base. **d** Optical cross section of inner tentacle near its tip. **e** The abfrontal tentacular nerve consists of two neurite bundles (violet), which are associated with the sensory cell (pink). **f** Numerous neurites of the frontal tentacular nerve. **g** Lateroabfrontal nerve of outer tentacle consists of neurites of different diameter with synaptic vesicles of different types. **h** Laterofrontal tentacular nerve. **i** Laterofrontal tentacular nerve consists of neurites with electron-lucent cytoplasm that contains thick microtubules (arrowheads). Abbreviations: afn – abfrontal tentacular nerve; ecm – extracellular matrix; gc – glandular cell; fn – frontal tentacular nerve; lafn – lateroabfrontal tentacular nerve; lfn – laterofrontal tentacular nerve; mi – microvilli; mc – muscle cell; n – nucleus; nf – nerve fiber; pn – peritoneal neurite; sv – synaptic vesicle

Cross sections of the **outer tentacles** reveal six tentacular nerves: abfrontal, two lateroabfrontal, two laterofrontal, and frontal (Figs. 5a, b; 11a, b).

The abfrontal tentacular nerve of the outer tentacle arises from the branches of adjacent intertentacular nerves. The abfrontal tentacular nerve extends along the abfrontal side of each tentacle and is represented by two thin bundles. The abfrontal neurite bundles are distant from each other at the base of the tentacle but are near each other at the tip. According to TEM, each neurite bundle consists of 5–12 nerve fibers (Figure 11e). Some of these nerve fibers belong to the cells, which are part of the abfrontal epithelium (Figure 11e). The lateroabfrontal tentacular nerve arises from the paired outer radial nerves. This nerve consists of three neurite bundles at the base of each tentacle (Figure 11a) and one neurite bundle at the tip (Figure 11b). According to TEM, each bundle consists of 10–15 nerve fibers and usually includes large-diameter neurites with many synaptic vesicles that contain electron-dense and electron-lucent material (Figure 11g). The lateroabfrontal tentacular nerves are associated with glandular cells that are scattered along the lateroabfrontal sides of the outer tentacles (Figs. 2c, 4a, 11g). The laterofrontal tentacular nerve arises from the second accessory nerve. Each laterofrontal tentacular nerve has a large diameter and is formed by 40–50 neurites of equal diameter (Figure 11h). These neurites contain thick microtubules that are scattered in an electron-lucent cytoplasm (Figure 11i). The frontal tentacular nerve arises from branches of the cross nerve. Each frontal tentacular nerve looks like a wide aggregation of neurites (Fig. 11a, b). According to TEM (Figure 11f), the frontal nerve includes more than 100 nerve fibers with different diameters and with synaptic vesicles that differ in content. These neurites are grouped into thicker central neurite bundles and also into several thinner peripheral neurite bundles (Figure 11a).

The innervation of the **inner tentacles** is similar to that of the outer tentacles especially with regard to the ultrastructure of the neurite bundles. There are some differences, however, in the sites of nerve origin and in the number of tentacular nerves. Cross sections of each inner tentacle reveal four tentacular nerves: abfrontal, two laterofrontal, and frontal (Fig. 11c, d).

The abfrontal tentacular nerve originates from the second accessory nerve. Each abfrontal nerve is represented by four neurite bundles (Fig. 11c, d). The laterofrontal tentacular nerves arise from the second accessory nerve; each laterofrontal nerve has a diameter of about 1–2 μm and is represented by an aggregation of neurites of specific ultrastructure. The frontal tentacular nerve arises from the branch of the cross nerve. Each frontal nerve is represented by a large aggregation of neurites with a prominent central portion (Figure 10c).

Tentacles of both inner and outer rows are innervated by **peritoneal neurites**, which exhibit strong immunoreactivity against acetylated alpha tubulin (Fig. 4c, d). These neurites extend longitudinally under the coelomic lining and form an inner “cylinder” in each tentacle (Figs. 4a, b; 10c). Peritoneal neurites start from cells (peritoneal perikarya) that exhibit strong immunoreactivity against acetylated alpha tubulin and are mostly located at the base of each tentacle and are also scattered along the tentacles (Fig. 4c, g). Peritoneal perikarya have electron-light cytoplasm and can be distinguished from typical peritoneal cells that have electron-dense cytoplasm (Figure 4c). Electron-light cytoplasm is characteristic of the preritoneal neurites as well (Figure 4h). Peritoneal neurites and peritoneal perikarya exhibit serotonin-like immunoreactivity (Figure 4e).

## Discussion

### Innervation of the lophophore in brachiopods

Brachiopods are distributed across three major groups: Linguliformea, Craniiformea, and Rhynchonelliform [12, 13].

The innervation of the lophophore in adult brachiopods has mostly been studied by histological methods [16–19]. The detailed descriptions of the nervous system of the brachiopod lophophore have been done for *Novocrania anomala*, *Discinisca lamellosa*, *Lingula anatina* [16, 18, 19], and *Gryphus vitreus* [17]. The nervous system of the lophophore in *Lingula anatina* was recently studied by a combination of TEM, immunocytochemistry, and CLSM [10].

**Fig. 12:**
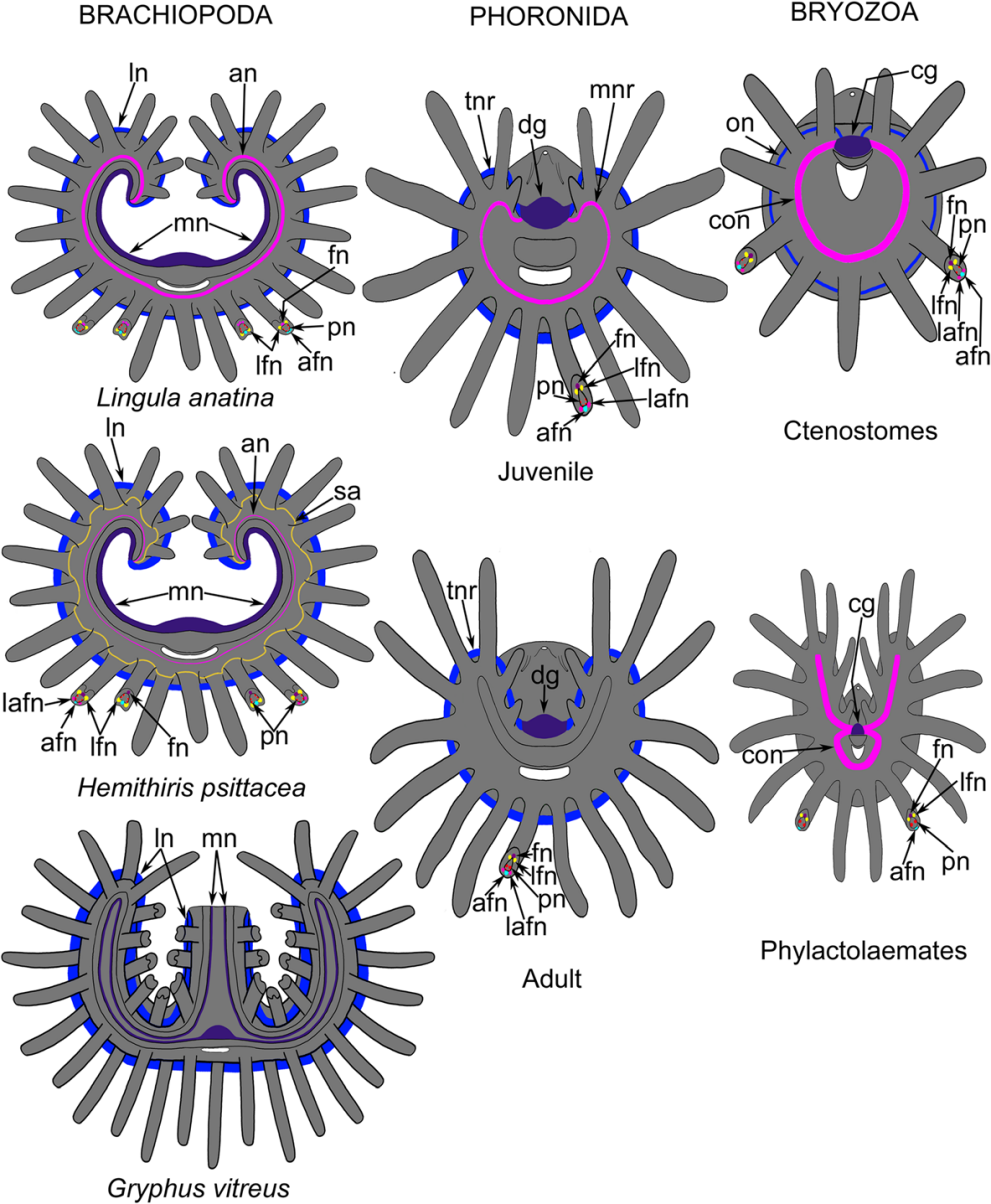
Schemes of innervation of the lophophore in different brachiopods, phoronids, and bryozoans. Structures that are thought to be homologous are marked by the same color. Among brachiopods, the inarticulate *Lingula anatina* has three brachial nerves: main, accessory, and lower. The accessory nerve is well developed and contributes to innervation of tentacles. All tentacles are innervated by four tentacular nerves: abfrontal, two laterofrontal, and frontal. The scheme is based on [10]. The rhynchonelliform *Hemithiris psittacea* has four brachial nerves: main, accessory, second accessory, and lower. The accessory brachial nerve is poorly developed and does not contribute to the innervation of tentacles, whereas the lower brachial nerve is stronger and more prominent than in *L. anatina*. In *H. psittacea*, the outer tentacles are innervated by six tentacular nerves: abfrontal, two lateroabfrontal, two laterofrontal, and frontal. The scheme is based on this report. The rhynchonelliform *Gryphus vitreus* has two brachial nerves: main and lower. The tentacles are innervated by the lower brachial nerve, which is well developed. Details of tentacle innervation are not known; the scheme is based on [17]. Among phoronids, juveniles have oval-shaped lophophore, three nerve elements of the lophophore: dorsal ganglion (dg), minor nerve ring (mnr), and tentacular main nerve ring (tnr), whereas adults have horseshoe-shaped lophophore and lack the minor nerve ring. In all phoronids, tentacles are innervated by six tentacular nerves: abfrontal, two lateroabfrontal, two laterofrontal, and frontal. Scheme of juvenile lophophore is based on [30]; scheme of adult lophophore is based on [31, 37]. Among bryozoans, ctenostome bryozoans have the cerebral ganglion (cg), circumoral nerve ring (con), and outer nerve ring (on), whereas many other bryozoans (i.e. phylactolaemates) lack the outer nerve ring. In bryozoans, tentacles are innervated by four tentacular nerves: abfrontal, two laterofrontal, and frontal. The scheme of ctenostome lophophore is based on [11]; the scheme of phylactolaemates is based on [33, 49]. Abbreviations: afn – abfrontal tentacular nerve; an – accessory brachial nerve; cg – cerebral ganglion; con – circumoral nerve ring; dg – dorsal ganglion; fn – frontal tentacular nerve; lafn – lateroabfrontal tentacular nerve; lfn – laterofrontal tentacular nerve; ln – lower brachial nerve; mn – main brachial nerve; mnr – minor nerve ring; pn – peritoneal neurite; sa – second accessory brachial nerve; tnr – tentacular main nerve ring

According to all these studies, the brachiopod lophophore has three nerves: the main brachial nerve, the accessory brachial nerve, and the lower brachial nerve (Fig. 12). The current data indicate that the lophophore of *H. psittacea*, in addition to containing these three nerves, also contains a second accessory nerve that has not been previously described in brachiopods (Fig. 12). The second accessory nerve in *H. psittacea* may be related to neurites in *L. anatina*, as indicated by the following observations. The base of the tentacles in *L. anatina* is usually penetrated by many neurites that contribute to the innervation of tentacles [23], and these neurite bundles extend at the base of tentacles exactly where the second accessory nerve is located in *H. psittacea*. In other words, the many neurites in *L. anatina* and the second accessory nerve in *H. psittacea* occur at that same location at the base of tentacles. This suggests that the second accessory nerve may represent an apomorphic step in the evolution of the lophophore nervous system of brachiopods. It may also be the case that the second accessory nerve belongs to the ground pattern of Brachiopoda but has been somewhat reduced in *L. anatina*.

According to their location with respect to the brachial fold and tentacles, the three nerves are apparently homologous in different species. On the other hand, the three main nerves contribute differently to the innervation of tentacles in different species. Thus, the main brachial nerve in *L. anatina* [10] and in *N. anomala* [18] gives rise to the cross nerves, which connect to the accessory brachial nerve. Tentacular innervation originates from the accessory brachial nerve. In *H. psittacea*, cross nerves are also present, but rather than connecting with the accessory brachial nerve, they extend directly into the tentacles. Thus, the main brachial nerve in *H. psittacea* directly contributes to the innervation of tentacles via cross nerves, and the accessory brachial nerve does not contribute to the innervation of tentacles at all. In the rhynchonelliform brachiopod *G. vitreus*, which is a species with a plectoploph lophophore (this type of lophophore is the most complex one within brachiopods), tentacles are innervated by the lower brachial nerve and not by the main brachial nerve [17, 24]. The reduced role of the main brachial nerve in the innervation of tentacles in *G. vitreus* may correlate with the organization of its plectolophe lophophore: for plectolophe lophophores, each arm of the lophophore is represented by a curve, in which the two curves are fused to each other along their inner sides where the main brachial nerve extends (Fig. 12).

The lower brachial nerve produces the abfrontal tentacular nerves of the outer tentacles in *L. anatina* [10] and in *H. psittacea* (herein). In both species, moreover, the lower brachial nerve gives rise to paired outer radial nerves, which extend to the tentacle bases and connect with aggregations of FMRFamide-like immunoreactive perikarya in *L. anatina* and with the second accessory nerve in *H. psittacea*. In the latter case, the lower brachial nerve contributes to the innervation of the lateroabfrontal zones of the outer tentacles via paired outer radial nerves, which give rise to the lateroabfrontal tentacular nerves. Although *L. anatina* and *H. psittacea* have the same type of the lophophore, the lower brachial nerve seems to contribute more to the innervation of tentacles in *H. psittacea* than in *L. anatina*. In the series from *L. anatina* to *H. psittacea* and to *G. vitreus,* an increasing contribution of the lower brachial nerve and a decreasing contribution of the accessory brachial nerve to the innervation of the tentacles is revealed (Figure 12).

### Innervation of tentacles in brachiopods

The innervation of tentacles in brachiopods has been seldom studied [19–21]. According to recent detailed results [10], the tentacles of *L. anatina* are innervated by four tentacular nerves: the frontal, two laterofrontal, and the abfrontal. The similarity in the innervation of different zones of the outer and inner tentacles of *L. anatina* suggests that the frontal zone is very wide in the inner tentacles but narrow in the outer tentacles [10]. The innervation of tentacles seems to be similar in *H. psittacea* and *L. anatina*. At the same time, there are some interesting differences. First, the frontal nerves of the inner tentacles in *L. anatina* arise from two adjacent intertentacular neurite bundles, whereas each inner tentacle in *H. psittacea* is innervated by one branch of the cross nerve. Second, the abfrontal nerve in each outer tentacle arises from adjacent intertentacular nerves in *H. psittacea* whereas the abfrontal zone of each outer tentacle is innervated by a group of neurites that arise from the lower brachial nerve in *L. anatina*. Third, the outer tentacles are innervated by six tentacular nerves in *H. psittacea* but by only four in *L. anatina*. In addition to having the typical four nerves, *H. psittacea* has two lateroabfrontal nerves. The presence of these additional nerves may correlate with the lateroabfrontal rows of abundant glandular cells in *H. psittacea*. The abundance of innervated glandular cells in *H. psittacea* may be related to the brooding of embryos and larvae, which stick to the lophophore [25–27]. Fourth, the tentacles of *H. psittacea* exhibit strong immunoreactivity against acetylated alpha tubulin, which is expressed by peritoneal neurites and perikarya. Although peritoneal neurites have been described in *L. anatina* [10], peritoneal neurites have not been shown to exhibit FMRFamide-like, serotonin-like, and acetylated alpha tubulin-like immunoreactivity.

### Innervation of the lophophore in the lophophorates

As noted earlier, the Lophophorata traditionally includes three phyla of invertebrates: brachiopods, phoronids, and bryozoans [1, 2]. Although the unity of the lophophorates has been criticized in many phylogenetic studies [4–7], the monophyly of the lophophorates has been supported by morphological data [10, 11] and few molecular studies [8, 9]. Because the lophophore is traditionally suggested as a homologous structure in all lophophorates, the comparative analysis of the nervous system of the lophophore and tentacles in the lophophorates may help clarify whether the lophophorates are monophyletic.

Previous analyses [10, 11] indicate that there are homologous nerves in the lophophores of brachiopods, phoronids, and bryozoans. According to its relative location to other homologous structures (tentacles, brachial fold/epistome, and mouth), the main brachial nerve of brachiopods is homologous to the dorsal nerve center of phoronids and to the cerebral ganglion of bryozoans. The accessory brachial nerve of brachiopods is homologous to the minor nerve ring of phoronids and to the circumoral nerve ring of bryozoans. The lower brachial nerve of brachiopods is homologous to the tentacle main nerve ring of phoronids and to the outer nerve ring that has been described in two ctenostome bryozoans [11].

Brachiopods exhibit different patterns of lophophore innervation, i.e., the development of the three main nerves and their contribution to the innervation of tentacles differ depending on the species. Because the relationships between large groups of brachiopods have not been strictly established [14, 28], it is impossible to determine which patterns of lophophore innervation are basal or derived. Determining which patterns are basal or derived is complicated by the finding that the morphology of adult rhynchonellides reflects successive juvenilization and that heterochrony may have played an important role in rhynchonellide evolution [29].

Although inarticulate brachiopods have recently been regarded as a sister group of phoronids [28], the lophophore innervation in phoronids has more in common with that of rhynchonelliform brachiopods than inarticulate brachiopods. The most evident similarity is the presence of the prominent lower brachial nerve and the less developed accessory brachial nerve. In adult phoronids, the tentacle main nerve ring is also very prominent, and the minor nerve ring is nearly absent. The minor nerve ring has been described in juvenile phoronids [30] (Figure 12) but not in adults [31, 32] (Figure 12). Interestingly, the opposite applies to bryozoans, i.e., the outer nerve ring is extremely weak and has been described only in ctenostomes (Figure 12). In other bryozoans, the outer nerve ring is absent, whereas the circumoral nerve ring is very thick (Figure 12).

### Innervation of tentacles in the lophophorates

According to published data concerning the organization of tentacles in the lophophorates [19, 20, 30, 32–41], there are several anatomical zones that extend along each tentacle. These zones differ in abundance of cilia and in the relative location to the mouth. Most lophophorates (phoronids, barchiopods, and bryozoans) studied to date have six zones, which are located along the tentacle perimeter: one frontal, one abfrontal, two laterofrontal, and two lateroabfrontal (or lateral). The frontal zone is the most heavily ciliated and faces the mouth. The laterofontal zones contain specific laterofrontal sensory cells and correspond to the postoral ciliated band [35, 36]. In phoronids and brachiopods, in addition to having choanocyte-like sensory cells, the laterofrontal zones are characterized by being innervated by specific tentacular nerves, which are formed by neurites that have large diameters, electron-lucent cytoplasm, and thick microtubules. This specific ultrastructure of neurites, which has been found in phoronids [42] and in brachiopods [10], suggests that the laterofrontal zones in these two groups are homologous.

The abfrontal zone in the lophophorates faces away from the mouth and has only a few cilia. The lateroabfrontal zones usually contain glandular cells in phoronids and brachiopods. As a rule, each zone is specifically innervated by tentacular nerves. Thus, six tentacular nerves typically extend along each tentacle. The number of tentacular nerves can exceed six, as is the case in phoronids [32, 39], or can be less than six, as is the case in bryozoans [11, 40, 41]. The difference in number of tentacular nerves may reflect some phylogenetic steps, which correlate with a decrease or increase in the number of tentacles and their size in different groups of lophophorates [11, 41].

All brachiopods studied to date have been described as having four zones of tentacles: frontal, two laterofrontal, and abfrontal [10, 19, 20]. These zones are characterized not only by a specific density of cilia but also by a specific ultrastructure of the peritoneal cells [20]. The presence of six groups of tentacular nerves in *H. psittacea* may reflect an ancestral state, which is characteristic of all lophophorates. On the other hand, the presence of lateroabfrontal tentacular nerves may correlate with presence of prominent lateroabfrontal rows of glandular cells in the outer tentacles of *H. psittacea*.

An interesting peculiarity of the tentacles in the lophophorates is the presence of subperitoneal neurites. The ultrastructure of subperitoneal neurites is similar in bryozoans [11, 31, 43], phoronids [36, 44], and brachiopods [10]. The strong immunoreactivity of subperitoneal neurites is described in this report for the first time and supports the neuronal nature of these structures, which was questioned in some papers [41].

### Are the lophophorates monophyletic?

New data on the nervous system organization in all groups of lophophorates may help indicate whether the lophophorates are monophyletic [45]. The presence of the lophophore—which is a part of the mesosome and which has tentacles and functions in respiration and food capture — is the most prominent feature in the lophophorates [1–3]. In all lophophorates, the tentacles of the lophophore surround the mouth but never the anus. It follows that entoprocts, whose tentacles surround both the mouth and the anus, cannot be regarded as lophophorates. Moreover, in entoprocts, there is no coelomic cavity in general and not in the tentacles, and tentacle musculature differs from that of lophophorates. Also, the ciliation of the tentacles and type of filtration are different in entoprocts and lophophorates.

Consideration of the lophophore allowed researchers to combine phoronids, brachiopods, and bryozoans into a united clade, the Lophophorata [1–3]. The unity of the Lophophorates has not been supported, however, by some recent molecular phylogenetic data [4–7]. According to these data, brachiopods and phoronids are close relatives and form the clade Brachiozoa, whereas bryozoans form a separate clade whose position in the phylogenetic tree is unclear. At the same time, fewer recent molecular phylogenetic studies support the monophyly of the lophophorates but not the unity of the Brachiozoa [8, 9]. Detailed morphological analyses, on the other hand, revealed the similarity in organization of the lophophore nervous system among different lophophorates [10, 11]. These morphological results argue for the homology of the lophophores in lophophorates and support the unity of the clade Lophophorata.

According to our new results, the lophophore has several main nerves that are homologous among different lophophorates. For example, the main brachial nerve extending along the dorsal side of the brachial fold of brachiopods is homologous to the dorsal ganglion of phoronids and to the cerebral ganglion of bryozons. The accessory brachial nerve passing along the frontal side of tentacles of brachiopods is homologous to the minor nerve ring of juvenile phoronids and to the circumoral nerve of bryozoans. The lower brachial nerve extending along the abfrontal side of tentacles of brachiopods is homologous to the tentacular nerve ring of phoronids and to the outer nerve of ctenostome bryozoans.

Although the general morphology of the lophophore and its nervous system looks very similar in the lophophorates, the location of tentacles and the zones where new tentacles are formed is different in brachiopods compared to phoronids and bryozoans. The macroscopic resemblance of the lophophore in bryozoans and phoronids is consistent with the idea that these groups are closely related [46, 47].

## Conclusions

This report provides the first data on the immunoreactivity of some nerve elements in the lophophore of the rhynchonelliform brachiopod *H. psittacea*. These new data suggest a scheme of innervation of the lophophore and tentacles in *H. psittacea*.

As in most brachiopods studied to date, the lophophore in *H. psittacea* is innervated by four longitudinal nerves: the main brachial nerve, the accessory brachial nerve, the second accessory brachial nerve, and the lower brachial nerve (Figure 12). In *H. psittacea*, the accessory brachial nerve does not contribute to the innervation of tentacles, whereas the lower brachial nerve and the second accessory nerve contribute greatly to tentacle innervation. The tendency for a reduced role of the accessory brachial nerve and an increased role of the lower brachial nerve increases in brachiopods from *L. anatina* to *H. psittacea* to *G. vitreus*. The same tendency can be found in phoronids, whose adults lack the minor nerve ring, which is a homolog of the accessory brachial nerve, but have a prominent tentacle nerve ring, which is a homolog of the lower brachial nerve. In bryozoans, the opposite is true: the outer nerve ring, which is a homolog of the lower brachial nerve, is very weak, whereas the circumoral nerve, which is a homolog of the accessory brachial nerve, is prominent.

The comparative analysis revealed that, among all lophophorates, lophophores with a simple organization (simple shape) have more main nerves than lophophores with a complex organization (Figure 12). Thus, the oval lophophore of juvenile phoronids is innervated by both the minor nerve ring and the tentacular nerve ring, whereas the horseshoe-shaped lophophore of adult phoronids is innervated only by the tentacular nerve ring (Figure 12-Phoronida). In bryozoans, the circular lophophore of ctenostomes has both outer and inner nerves, whereas the horseshoe-shaped lophophore of phylactolaemates has only the inner nerve (Figure 12-Bryozoa). In brachiopods, the simple spirolophe lophophore is innervated by main, accessory, second accessory, and outer nerves, whereas the complex plectolophe lophophore only has main and outer nerves (Figure 12-Brachiopoda). This inference is consistent with the view that the oval lophophore is the most primitive lophophore in phoronids [48]; that the circular lophophore might be the most primitive lophophore in bryozoans [49]; and that the trocholophe (or schizolophe) lophophore is the most primitive lophophore in brachiopods [2]. If a simple lophophore reflects the primitive state in each group of lophophorates, the complex innervation (the presence of many nerve elements), which is characteristic of the lophophore of simple shape, should be regarded as the ancestral (basal) state for all lophophorates. Thus, the presence of a cerebral ganglion and inner and outer nerves is the ancestral state of lophophore innervation in the lophophorates. According to this view, the absence of some nerve elements is the apomorphic (derived) state, which developed due to the reduction or fusion of some nerve elements in lophophorates with complex lophophores.

The innervation of tentacles in brachiopods seems to be similar among the different species studied to date. All species have at least four tentacular nerves: the frontal, two laterofrontal, and the abfrontal. The presence of additional two lateroabfrontal tentacular nerves in *H. psittacea* may reflect the ancestral state of the tentacle innervation in all lophophorates. On the other hand, the presence of two additional lateroabfrontal tentacular nerves in *H. psittacea* may correlate with the specific organization of tentacular glandular cells, whose contents are probably used to glue embryos and larvae to the lophophore in *H. psittacea*.

## Methods

### Animals

Adults of *Hemithiris psittacea* (Gmelin, 1791) were collected from May–August 2014–2015 in Kandalakshskii Bay of the White Sea, near the White Sea biological station of Moscow State University (66°33′1.84″N; 33° 7′47.19″E).

This study is focused on the lophophore of *H. psittacea*. Animals were photographed in the laboratory using a Leica M165C stereomicroscope equipped with a Leica DFC420 digital camera (Leica Microsystems GmbH, Wetzlar, Germany). The brachial valve of the shell was removed, and the specimens were dissected to obtain lophophore with mouth, tentacles, and brachial fold. Parts of the lophophores were fixed for semi-thin sectioning, scanning electron microscopy (SEM), TEM and CLSM.

### Microscopy

For detailed description of methods of electron microscopy and immunocytochemistry see [10]. For SEM, specimens were fixed in a 4% paraformaldehyde solution, dehydrated in ethanol followed by an acetone series, critical point dried, and then sputter coated with platinum-palladium alloy. Specimens were examined with a JEOL JSM scanning electron microscope (JEOL Ltd., Tokyo, Japan).

For semi-thin sectioning and TEM, specimens were fixed at 4 °C in 2.5% glutaraldehyde and were then postfixed in 1% osmium tetroxide, dehydrated in ethanol, and embedded in Spurr resin (Sigma-Aldrich, USA). Semi-thin and thin sections were prepared with a Leica UC6 ultramicrotome (Leica Microsystems GmbH, Wetzlar, Germany). Semi-thin sections were stained with methylene blue, observed with a Zeiss Axioplan2 microscope, and photographed with an AxioCam HRm camera (Carl Zeiss, Oberkochen, Germany). Thin sections were stained with lead citrate (0.4%) and then examined with a JEOL JEM 100B electron microscope (JEOL Ltd., Tokyo, Japan).

For immunocytochemistry, parts of *H. psittacea* lophophores were fixed in 4% paraformaldehyde solution, washed in phosphate buffer (pH 7.4) (Fisher Scientific, Pittsburgh, PA, USA) with Triton X-100 (2%) (Fisher Scientific) (PBT). Nonspecific binding sites were blocked with 10% normal donkey serum (Jackson ImmunoResearch, Newmarket, Suffolk, UK) in PBT. The specimens were incubated in primary antibody (anti-ɑ-Tubulin-mouse (1:700) and anti-FMRFamide-rabbit (1:800) or anti-serotonin-rabbit (1:1000) (ImmunoStar, Hudson, WI, USA) in phosphate buffer with Triton X-100), washed in PBT, exposed to the secondary antibody (532-Alexa-Rabbit (1:1000) and 635-Alexa-Mouse (1:1000) (Invitrogen, Grand Island, NY, USA)), washed in phosphate buffer, embedded in Murray Clear, mounted on a glasses covered with poly-L-lysine (Sigma-Aldrich, St. Louis, MO, USA), and examined with a Nikon Eclipse Ti confocal microscope (Moscow State University, Moscow, Russia). Z-projections were done using Image J version 1.43 software. Volume rendering were prepared using Amira version 5.2.2 software (Thermo Fisher Scientific, MA, USA). TEM micrographs and Z-projections were processed in Adobe Photoshop CS3 (Adobe World Headquarters, San Jose, California, USA) to prepare panoramas and combinations of Z-projections.

## Abbreviations

CLSM: Confocal laser scanning microscopy
PBT: Phosphate buffer with Triton X-100
SEM: Scanning electron microscopy
TEM: Transmission electron microscopy
